# Acoustically induced coherent spin trapping

**DOI:** 10.1126/sciadv.abj5030

**Published:** 2021-10-29

**Authors:** Alberto Hernández-Mínguez, Alexander V. Poshakinskiy, Michael Hollenbach, Paulo V. Santos, Georgy V. Astakhov

**Affiliations:** 1 Paul-Drude-Institut für Festkörperelektronik, Leibniz-Institut im Forschungsverbund Berlin e.V., Hausvogteiplatz 5-7, 10117 Berlin, Germany.; 2 Ioffe Physical-Technical Institute, Russian Academy of Sciences, 194021 St. Petersburg, Russia.; 3 Helmholtz-Zentrum Dresden-Rossendorf, Institute of Ion Beam Physics and Materials Research, Bautzner Landstrasse 400, 01328 Dresden, Germany.; 4 Technische Universität Dresden, 01062 Dresden, Germany.

## Abstract

Spin centers are promising qubits for quantum technologies. Here, we show that the acoustic manipulation of spin qubits in their electronic excited state provides an approach for coherent spin control inaccessible so far. We demonstrate a giant interaction between the strain field of a surface acoustic wave (SAW) and the excited-state spin of silicon vacancies in silicon carbide, which is about two orders of magnitude stronger than in the ground state. The simultaneous spin driving in the ground and excited states with the same SAW leads to the trapping of the spin along a direction given by the frequency detuning from the corresponding spin resonances. The coherence of the spin-trapped states becomes only limited by relaxation processes intrinsic to the ground state. The coherent acoustic manipulation of spins in the ground and excited state provides new opportunities for efficient on-chip quantum information protocols and coherent sensing.

## Teaser

Simultaneous strain-driven transitions in ground and excited state of spin qubits lead to the coherent trapping of the spin state.

## INTRODUCTION

Hybrid spin-optomechanical quantum systems offer high flexibility, integrability, and applicability for quantum science and technology (*1*–*10*). Particularly, on-chip surface acoustic waves (SAWs) (*11*) can efficiently drive spin transitions of atomic-scale, color-center qubits, which are forbidden in case of the more frequently used electromagnetic fields (*12*–*14*). Compared to the ground states, their optically accessible excited states have even stronger interaction with strain fields, giving rise to their polaronic character (*15*), giant thermal shift (*16*), and Landau-Zener-Stückelberg interference (*17*). These properties give rise to novel and, so far, largely unexploited physical phenomena. As an example, the electron-phonon coupling between the strain fields of SAWs and the orbital degrees of freedom in the excited state has been used to realize phonon-assisted optical transitions (*18*), as well as optically driven spin transitions and coherent population trapping (*19*).

In this contribution, we first show that the strength of the SAW-induced spin transitions within the excited-state spin sublevels of the silicon vacancy center in SiC exceeds by about two orders of magnitude of that of the ground state. We then take advantage of this giant spin-phonon coupling to realize simultaneous strain-driven transitions between spin sublevels in both the ground and excited state. We demonstrate that the interference between these spin transitions induced by a single SAW leads to an acoustically induced coherent spin trapping (CST) in states with polarization fixed along a well-defined direction, which bears conceptual analogies to coherent population trapping and electromagnetically induced transparency (*20*). The coherence time of the CST is insensitive to the optical transitions and is only limited by intrinsic relaxation processes in the ground state.

**Fig. 1.**
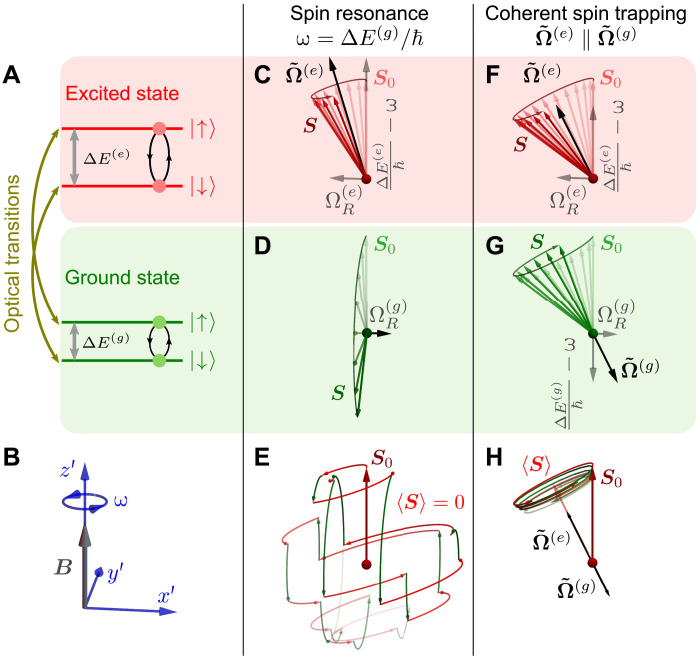
Comparison of conventional spin resonance and CST. (**A**) Electronic-level structure of the ground and excited state of the spin-1/2 center. The black cycles represent spin transitions driven by an ac field at the frequency ω. Yellow green arrows show spin-preserving optical transitions between the ground and excited states. (**B**) The reference frame rotating around the spin quantization axis *z*′ with the frequency ω where the spin dynamics is analyzed. (**C**, **D**, **F**, and **G**) Spin dynamics in the ground and excited states in the rotating frame under conventional spin resonance and CST, respectively. (**E**) In case of spin resonance, the precession frequencies in the ground and excited states are not aligned and the spin dephases rapidly after a few optical transitions. (**H**) Under CST, the precession frequencies are aligned and the spin projection along this direction is conserved independently of the optical transitions between ground and excited state.

To describe the CST, we consider a two-level spin system (spin up and spin down) under a static magnetic field **B** with different ground-state and excited-state splittings, indicated in Fig. 1A as Δ*E*^(*g*)^ and Δ*E*^(*e*)^, respectively. An oscillating field of angular frequency ω drives the precession between the spin-up and spin-down directions (Rabi oscillations) in both ground and excited states. The spin dynamics under the driving field are analyzed in the rotating wave approximation. In the reference frame that rotates with the driving frequency ω around the spin quantization axis $\hat{z}\prime$ (Fig. 1B), the Rabi oscillations can be conveniently represented in a vectorial form, where the spin precesses around an effective field ${\tilde{\Omega }}^{\left(g\right)}$ in the ground state and a different effective field ${\tilde{\Omega }}^{\left(e\right)}$ in the excited state.

If the driving frequency is equal to the ground-state spin resonance frequency ω = ħ^−1^Δ*E*^(*g*)^, then the spin in the ground state precesses around a transverse (i.e., $∥\hat{x}\prime$) effective field ${\tilde{\Omega }}^{\left(g\right)}=\Omega_{R}^{\left(g\right)}\hat{x}\prime$, with the Rabi frequency $\Omega_{R}^{\left(g\right)}$ (see Fig. 1D). The latter is proportional to the amplitude of the driving field following $\hbar \Omega_{R}^{\left(g\right)}=g^{\left(g\right)}\mu_{B}b_{\text{rf}}$ for radiofrequency (RF) driving or $\hbar \Omega_{R}^{\left(g\right)}=\Xi^{\left(g\right)}\varepsilon$ for acoustic driving (as in this work). Here, ħ is the reduced Planck constant, *g*^(*g*)^ is the ground-state *g*-factor, μ*_B_* is the Bohr magneton, *b*_rf_ is the amplitude of the RF magnetic field, Ξ^(*g*)^ is the spin-strain interaction constant in the ground state, and ε is the acoustically induced strain.

Because Δ*E*^(*g*)^ ≠ Δ*E*^(*e*)^, the driving frequency is detuned from the resonance frequency in the excited state. In this case, ${\tilde{\Omega }}^{\left(e\right)}$ consists of a transverse and a longitudinal (i.e., ∥ $\hat{z}$′) component (*21*)

$${\tilde{\Omega }}^{\left(e\right)}=\Omega_{R}^{\left(e\right)}\hat{x}\prime +(\hbar^{-1}\Delta E^{\left(e\right)}-\omega )\hat{z}\prime$$ (1)

where $\Omega_{R}^{\left(e\right)}$ is the Rabi frequency in the excited state. Therefore, the Rabi oscillations in the excited state will take place at the generalized Rabi frequency ${\tilde{\Omega }}^{\left(e\right)}={\left[{\left(\Omega_{R}^{\left(e\right)}\right)}^{2}+{\left(\hbar^{-1}\Delta E^{\left(e\right)}-\omega \right)}^{2}\right]}^{1/2}$ and around a different precession axis than in the ground state (see Fig. 1C).

Under a continuous optical excitation, the spin switches randomly between the ground and excited states. Here, we will assume that the spin orientation is preserved during the optical transitions (see yellow green arrows in Fig. 1A). The random switching between ground and excited states leads to random changes between ${\tilde{\Omega }}^{\left(g\right)}$ and ${\tilde{\Omega }}^{\left(e\right)}$ (and, therefore, of the spin precession frequency and direction). This results in a fast spin dephasing (Fig. 1E), which is analogous to the Dyakonov-Perel spin relaxation mechanism (*22*).

A different situation emerges if one detunes the frequency ɷ of the driving field from the ground state resonance in such a way that both ${\tilde{\Omega }}^{\left(g\right)}$ and ${\tilde{\Omega }}^{\left(e\right)}$ have non-zero longitudinal and transverse components, but turn out to be collinear (as in Fig. 1, F and G)—the CST condition. Here, ${\tilde{\Omega }}^{\left(g\right)}$ is also given by Eq. 1, with the superscript (*e*) replaced by (*g*). Then, the collinearity condition can be written as

$$\frac{\hbar^{-1}\Delta E^{\left(g\right)}-\omega }{\hbar^{-1}\Delta E^{\left(e\right)}-\omega }=\frac{\Omega_{R}^{\left(g\right)}}{\Omega_{R}^{\left(e\right)}}$$ (2)

and CST occurs for the driving frequency

$$\omega_{\text{CST}}=\frac{\Delta E^{\left(g\right)}\Omega_{R}^{\left(e\right)}-\Delta E^{\left(e\right)}\Omega_{R}^{\left(g\right)}}{\hbar (\Omega_{R}^{\left(e\right)}-\Omega_{R}^{\left(g\right)})}$$ (3)

Under this condition, the spin precession axis is no longer affected by the optical transitions between the ground and excited state, and thus, the spin projection on this axis remains conserved for times limited only by intrinsic spin relaxation processes (Fig. 1H) (*23*–*25*). This coherent trapping of the spin polarization is analogous to the mechanism of coherent population trapping (*19*, *20*) in the sense that, in both cases, the system can be understood as a set of coupled oscillators that become effectively decoupled from one of their degrees of freedom (i.e., the optical transitions).

Following our theoretical considerations, the CST condition can always be achieved for the general case of Δ*E*^(*e*)^ ≠ Δ*E*^(*g*)^ provided that the Rabi frequencies in the ground and excited states are distinct, that is, $\Omega_{R}^{\left(g\right)}\neq \Omega_{R}^{\left(e\right)}$ (compare Eq. 2). If the spin system has the same *g*-factor for the ground and excited states (*26*), then this requirement cannot be fulfilled using RF magnetic fields because $\hbar \Omega_{R}^{\left(g\right)}=\hbar \Omega_{R}^{\left(e\right)}=g\mu_{B}b_{\text{rf}}$. In contrast, if the driving field is the oscillating strain ε, then the different extension of the electron wave function in the ground and excited states leads typically to different spin-strain interaction constants Ξ^(*g*)^ ≠ Ξ^(*e*)^ (*15*, *16*, *27*) and, therefore, to different Rabi frequencies $\hbar \Omega_{R}^{\left(g,e\right)}=\Xi^{\left(g,e\right)}\varepsilon$, so that CST can be realized experimentally.

## RESULTS

**Fig. 2.**
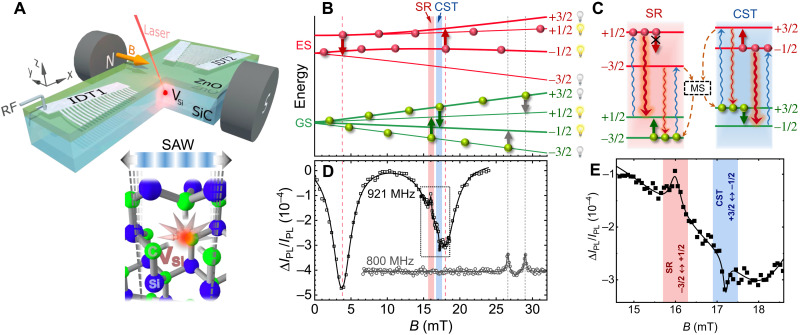
Ground-state and excited-state spin-acoustic resonances. (**A**) Acoustic resonator consisting of two focusing interdigital transducers (IDTs) exciting a standing SAW oscillating perpendicularly to an in-plane magnetic field *B*. The *V*_Si_ centers (inset) created below the 4H-SiC surface are optically pumped by a laser; the excited photoluminescence intensity is linked to the occupation of the *V*_Si_ spin sublevels. (**B**) Evolution of the spin sublevels in the ground state (GS) and excited state (ES) with external magnetic field at 125 K. The green and red vertical arrows indicate the acoustically induced spin transitions with Δ*m* = ± 2 at the SAW frequency of 921 MHz; the gray arrows denote the RF-induced spin transitions measured at 800 MHz. (**C**) Scheme of optical initialization and readout of the spin state. Optical pumping (blue curly arrows) together with nonradiative spin-dependent recombination (dashed arrows) via a metastable state (MS) lead to the preferential population of the spin states with *m_S_* = ± 3/2 in the GS (green circles) and *m_S_* = ±1/2 in the ES (red circles). The photoluminescence intensity is stronger for optical transitions between the *m_S_* = ±1/2 spin states [see red curly arrows and on/off light bulbs in (B)]. (**D**) Black squares show an optically detected spin resonance spectrum recorded at 125 K as a function of the external magnetic field for ω_SAW_/2π = 921 MHz. The solid line is a fit as described in the text. Gray circles display an optically detected spin resonance recorded for an RF signal ω/2π = 800 MHz applied to the IDT. The spectra are vertically shifted for clarity. (**E**) Close-up of the optically detected spin resonance spectrum around the magnetic field region of the GS resonances [dotted square in (D)].

To observe the proposed CST phenomenon, we make use of the so-called V2 center in 4H-SiC (Fig. 2A), a silicon vacancy (*V*_Si_) with spin *S* = 3/2 and well-studied optically detected spin resonances in external magnetic fields (*28*). We created an ensemble of *V*_Si_ centers at a mean depth of about 2.5 μm below the sample surface by proton irradiation (Materials and Methods) (*29*). The *V*_Si_ spins are driven by the dynamic strain field of a SAW resonator fabricated on top of the samples (Materials and Methods), as schematically shown in Fig. 2A. The in-plane magnetic field **B** = (0, *B_y_*,0), applied perpendicular to the SAW propagation direction, brings the transition frequencies between the spin sublevels of the *V*_Si_ centers into resonance with the SAW frequency ω_SAW_/2π.

Figure 2B displays the magnetic field dependence of the spin sublevels in the ground and excited states, calculated from the corresponding effective spin Hamiltonians in the uniaxial approximation

$${ℋ}^{\left(g,e\right)}=D^{\left(g,e\right)}(S_{z}^{2}-\frac{5}{4})+g^{\left(g,e\right)}\mu_{B}B\cdot S$$ (4)

Here, **S** = (*S_x_*, *S_y_*, *S_z_*) is the spin-3/2 operator, *g*^(*g*)^ = *g*^(*e*)^ ≈ 2 (*26*, *30*), and *D*^(*g*, *e*)^ are the zero-field splitting constants stemming from the crystal field. Under zero magnetic field, the spin sublevels are split at room temperature (RT) into two Kramer’s doublets with a separation of 2*D*^(*g*)^ = 70 MHz and 2*D*^(*e*)^ = 430 MHz between the states with the spin projections *m_S_* = ±1/2 and *m_S_* = ±3/2 on the hexagonal axis $\hat{z}$ (perpendicular to the sample plane) (*31*). For strong fields (i.e., *g*μ*_B_* ∣*B*∣ ≫ ∣*D*^(*e*, *g*)^∣, the spin projection becomes quantized along the magnetic field direction $\hat{y}$, and all spin sublevels are split. Because the direction of **B** and the SAW propagation direction are perpendicular to each other, the dynamic strain of the SAW resonator only drives spin transitions with Δ*m_S_* = ±2 (*14*) (see green and red vertical arrows in Fig. 2B). Therefore, this four-level spin system can be effectively separated in two decoupled two-level systems, as displayed in Fig. 2C (compare Fig. 1A).

The acoustically driven spin resonances are detected by recording changes in the photoluminescence intensity, *I*_PL_, as a function of the external magnetic field for a fixed ω_SAW_ (*14*). Figure 2C shows a summary of the optical initialization and readout of the *V*_Si_ spin polarization. The allowed optical transitions between the ground and excited spin sublevels are restricted to the ones indicated by the curly arrows because of the spin conservation condition typical of this spin center (*26*). The excited state also relaxes nonradiatively (dashed arrows) through spin-dependent recombination via a metastable state enabled by a combination of spin-orbit coupling and interaction with vibrational modes (*32*), leading to the preferential population of the spin sublevels with *m_S_* = ±3/2 in the ground state and *m_S_* = ±1/2 in the excited state [compare circles in Fig. 2 (B and C)]. The latter depends on the excitation conditions (*31*, *33*), a detailed phenomenological model is presented in the Supplementary Materials. As a consequence of the spin-selective intersystem crossing via the metastable state, the photoluminescence intensity is stronger for the *m_S_* = ±1/2 sublevels than for the *m_S_* = ±3/2 ones (*34*) (compare the thickness of the curly arrows in Fig. 2C and the on/off light bulbs in Fig. 2B). Therefore, an increase (decrease) of *I*_PL_ is expected when the SAW drives transitions between the spin sublevels in the ground (excited) state.

Figure 2D shows an optically detected spin resonance spectrum (open squares) at *T* = 125 K in the magnetic field range where the acoustically driven spin transitions with Δ*m_S_* = ±2 take place (*9*, *14*). Under excitation by a ω_SAW_/2π = 921 MHz SAW (see the Supplementary Materials for the characterization of the acoustic resonator), we observe two broad dips at *B* = 4 mT and *B* = 18 mT. They are associated with the excited-state spin transitions (+1/2 → −3/2) and (−1/2 → +3/2), respectively (compare red vertical arrows in Fig. 2B). In addition, the spectrum shows a narrow peak at *B* ≈ 16 mT and a narrow dip at *B* ≈ 17 mT. Figure 2E shows a close-up of the resonance spectrum in this magnetic field range. The narrow peak corresponds to the conventional spin resonance (marked as SR in the figure) attributed to the ground-state (−3/2 → +1/2) spin transition, as displayed in Fig. 2C. In contrast, the narrow dip cannot be described by the conventional ground-state spin resonance. Below, we show that it arises from the interference between the ground- and excited-state (+3/2 ↔ −1/2) spin transitions leading to the CST (see Fig. 2C). As shown in the Supplementary Materials (see eq. S17), the areas of the spin resonances are proportional to the spin transition rate multiplied by the average time that the center spends in the corresponding ground or excited state. Taking into account that the areas of the broad dips are ∼50 times larger than the areas of the narrow peak and dip and considering that the experiment is performed in the regime of weak optical pumping (that is, the spin stays a much longer time in the ground than in the excited state), we estimate the acoustically induced spin transition rate in the excited state to be, at least, 50 times stronger than in the ground state.

To verify the acoustic nature of the spin transitions, we carried out optically detected spin resonance experiments driven by an RF field (ω/2π = 800 MHz) below the resonance frequency of the acoustic resonator and, therefore, in the absence of an acoustic field (open circles in Fig. 2D). The changes in luminescence contrast only show the Δ*m_S_* = ±1 spin resonances purely driven by the RF magnetic field (compare gray vertical arrows in Fig. 2B), thus confirming that the peak and dips associated with the Δ*m_S_* = ±2 spin transitions can only be excited by the dynamic SAW strain field.

**Fig. 3.**
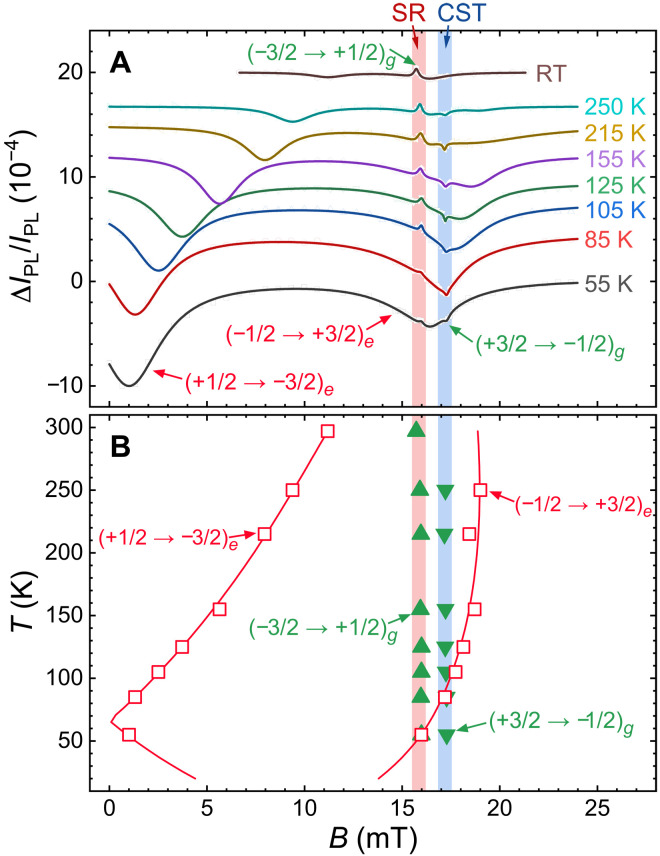
Temperature dependence of the spin-acoustic resonance. (**A**) Optically detected spin resonance spectra for different temperatures. The solid curves are fits to Eq. 5. The data are vertically shifted for clarity. (**B**) Solid triangles and open squares show the magnetic field positions *B_j_* of the Δ*m_S_* = ±2 ground- and excited-state spin transitions, respectively, obtained from the fits. Calculations for the excited-state transitions are represented by the red lines.

The most remarkable (and unexpected) feature in the acoustically driven spin resonance spectrum of Fig. 2D is the negative sign of the ground-state (+3/2 → −1/2) spin transition at *B* ≈ 17 mT (note that, according to the preferential population of the spin states, the sign of the resonance should be positive, as for the spin transition at *B* ≈ 16 mT). Detailed studies presented below reveal that it even has a more complex, Fano-like shape (*35*). This behavior is a fingerprint of CST. To further confirm this assumption, we measured acoustically induced spin resonance spectra at different temperatures (see Fig. 3). While the ground-state zero-field splitting 2*D*^(*g*)^ is almost temperature independent, the excited-state zero-field splitting 2*D*^(*e*)^ increases at a rate of 2.1 MHz/K with reducing temperature (*16*), thus shifting the spin resonances of the excited state toward lower magnetic fields (Fig. 3A).

We fit the experimental curves by a sum of Fano-like resonance functions (*35*)

$$\Delta I_{\text{PL}}/I_{\text{PL}}=\sum_{j}\frac{A_{j}\delta B_{j}^{2}+Q_{j}(B-B_{j})\delta B_{j}}{{\left(B-B_{j}\right)}^{2}+\delta B_{j}^{2}}$$ (5)

where the summation goes over all the spin transitions in the ground and excited states. Here, *B_j_* denote the magnetic field positions of the resonances, *δB_j_* are their widths, and *A_j_* and *Q_j_* are the amplitudes of the symmetric and antisymmetric parts of the resonances. The values of *B_j_* are depicted in Fig. 3B, together with the theoretical temperature dependences for the excited-state resonances (the red lines in Fig. 3B) obtained from the spin Hamiltonian in Eq. 4 and the known temperature dependence of 2*D*^(*e*)^ (*16*). The temperature variation allows direct monitoring of the CST as a function of the detuning between the excited-state (−1/2 → +3/2) spin transition and its ground-state (+3/2 → −1/2) counterpart. In parallel, the other excited-state spin resonance (+1/2 → −3/2) is strongly detuned from its ground-state (−3/2 → +1/2) counterpart for all temperatures. The latter behaves as a normal spin resonance and, hence, can be used as a reference.

**Fig. 4.**
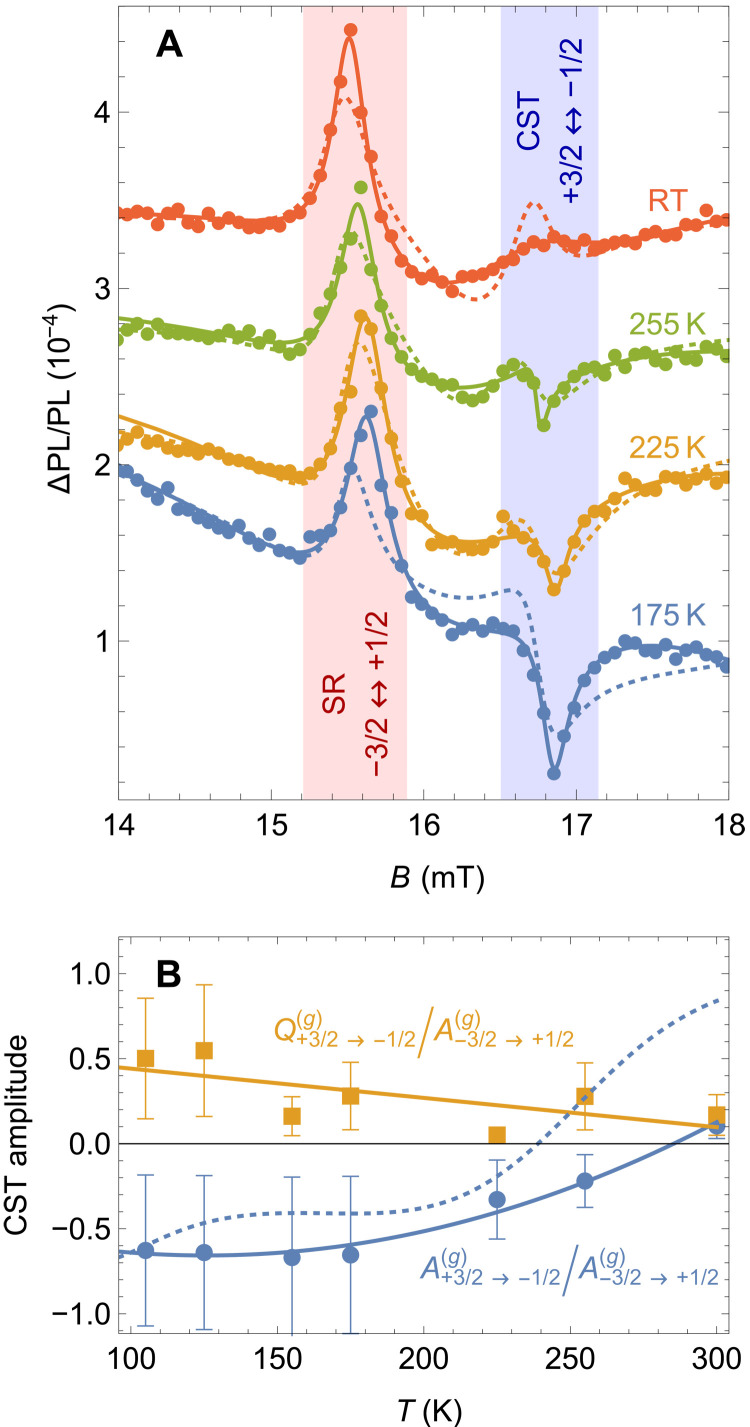
CST for different detunings. (**A**) Detailed measurement of the Δ*m_S_* = ±2 ground-state spin resonance spectra at representative temperatures. The solid lines are fits to Eq. 5. The dashed lines are calculations based on our microscopic model. The data are vertically shifted for clarity. (**B**) Parameters of the (+3/2 → −1/2) resonance as a function of temperature. Amplitudes of the symmetric and antisymmetric parts of the resonance line are shown. The values are normalized by the amplitude of the (−3/2 → +1/2) spin resonance. The solid lines are a guide for eye, and the dashed line is calculated after the model.

Detailed temperature dependences of the ground-state Δ*m_S_* = ±2 spin resonances are presented in Fig. 4A. At RT, both resonances appear as positive peaks in the SAR spectrum, but the ( +3/2 → −1/2) spin resonance at 17 mT is almost suppressed because of the CST mechanism. As the temperature decreases and the detuning between the (+3/2 ↔ −1/2) spin resonances in the ground and excited states is reduced, the spin transition at 17 mT becomes asymmetric and eventually changes its sign. The fits by Eq. 5 are in very good agreement with our experimental data (solid lines in Fig. 4A). We find that *Q_j_* = 0 for all resonances except for the ground-state ( + 3/2 → −1/2) spin transition, indicating its asymmetric Fano-like shape. We attribute this exceptional feature to the interference with the closely located excited-state (−1/2 → +3/2) spin resonance leading to the CST. Figure 4B shows the temperature dependence of the symmetric and antisymmetric parts of the resonance at 17 mT, $A_{+3/2\to -1/2}^{\left(g\right)}$ (blue circles) and $Q_{+3/2\to -1/2}^{\left(g\right)}$ (yellow squares). We normalized them by the amplitude of the other ground-state resonance $A_{-3/2\to +1/2}^{\left(g\right)}$, which remains always positive because its excited-state counterpart (+1/2 → −3/2) is far detuned. The negative amplitude of $A_{+3/2\to -1/2}^{\left(g\right)}$ at low temperatures originates from the interference of the ground- and excited-state (+3/2 ↔ −1/2) spin transitions. As the temperature increases, its value goes to zero at around 280 K and then becomes slightly positive, reflecting the suppression of the interference due to the increase of the detuning between the resonances and the speedup of the decoherence processes at high temperatures. The antisymmetric contribution to the resonance is always present, but it becomes comparable to the symmetric one at *T* ≈ 250 K, giving rise to the strongly asymmetric line shape.

## DISCUSSION

To account for the magnetic field dependencies, we develop a microscopic model, which includes the spin sublevels in the ground and excited states, together with an intermediate metastable state (Supplementary Materials) (*34*). As shown in Fig. 2C, we take into account spin-conserving optical transitions between the ground and excited states, and a spin-dependent transition rate to the metastable state, which enables spin polarization under optical pumping and explains the *I*_PL_ dependence on the spin polarization degree. Our model predicts that far-detuned resonances between the same spin sublevels in the ground and excited states should lead to the positive and negative sign of the acoustically induced spin resonance, respectively (Supplementary Materials), in agreement with the ground-state (−3/2 → +1/2) and excited-state (+1/2 → −3/2) spin resonances in the experimental spectra. However, if the detuning becomes comparable to the width of the excited-state spin resonance, then the ground-state spin resonance takes an asymmetric line shape and eventually flips its sign. The dashed lines in Fig. 4 (A and B) display the calculated spin resonance spectra and the resonance amplitudes following this model. They show a good quantitative agreement with our experimental data and reveal all essential features of the CST mechanism.

Another prediction of the model is the increase of the spin lifetime because of the CST phenomenon. In the CST condition, the spin dynamics gets decoupled from the optical transitions, thus suppressing the dominant spin relaxation mechanism. This should lead to a marked decrease of the ground-state resonance width from a large value determined by the optical pump rate to a much smaller value determined by the intrinsic spin relaxation rate (see eqs. S39 to S41). However, this resonance narrowing is masked in spin ensembles by the inhomogeneous broadening originated from fluctuations of the zero-field splittings, nuclear fields, and the magnitude of the strain field within the sample volume excited by the laser. It should be possible to observe the CST-induced resonance narrowing in experiments involving single-spin centers, but this is out of the scope of this manuscript.

In conclusion, we have demonstrated that SAWs can efficiently control transitions in atomic-scale spin centers both in the ground and excited state. Their simultaneous driving with the same SAW field leads to the coherent trapping of the spin polarization along a well-defined direction. It manifests itself as a suppression of the spin relaxation compared to a canonical spin resonance under the same conditions. We have developed a microscopic model for the acoustically induced CST, which shows a good quantitative agreement with the experimental data. The present demonstration was done using a single SAW resonator and nonresonant optical excitation, which addresses an inhomogenous ensemble of spin centers. Bichromatic excitation (hole-burning technique) (*36*) using a pair of SAW resonators or resonant optical excitation could be used to address a homogeneous ensemble of spin centers. This would markedly decrease the linewidth of the resonances and make the CST condition highly perspective for coherent sensing applications (*1*–*3*, *9*).

Besides CST, acoustic excitation of both ground and excited state opens a plethora of new ways for coherent spin control. In combination with the double RF-optical resonance (*37*) and fast reconfigurable quantum emitters (*17*), our approach can be extended for the control of individual spin qubits with coherent acoustic and optical fields. The acoustic driving approach is also promising for the implementation of quantum transducers (*4*), mechanical cooling (*5*), and photon (*8*) or phonon (*7*) networks; all these represent milestones toward on-chip quantum information processing on different material platforms.

## MATERIALS AND METHODS

### Sample fabrication

The *V*_Si_ centers were created in a 10 mm by 10 mm semi-insulating 4H-SiC substrate by the irradiation with protons with an energy of 375 keV and a fluence of 10^15^ cm^−2^. After irradiation, the SiC substrate was coated with a 35-nm-thick SiO_2_ layer followed by a 700-nm-thick ZnO piezoelectric film using RF magnetron sputtering. Acoustic resonators defined by a pair of focusing interdigital transducers (IDTs) were then patterned on the surface of the ZnO film by electron beam lithography and metal evaporation. Each IDT consists of 80 aluminum finger pairs for the excitation/detection of SAWs with a wavelength λ_SAW_ = 6 μm. An additional acoustic Bragg reflector consisting of 40 finger pairs was placed on the IDT’s backside. The finger curvature and separation between the opposite IDTs (≈120 μm) are designed to focus the SAW beam at the center of the resonator.

### Measurements

The optically detected spin-acoustic resonance experiments were performed in a confocal microphotoluminescence setup. The sample was placed in a cold-finger cryostat equipped with a window for optical access and coaxial connections for the application of the RF signals to the IDTs. The spin transitions in the *V*_Si_ centers were tuned to the frequency of the SAW by an in-plane magnetic field applied using an electromagnet. The *V*_Si_ centers were optically excited by a 780-nm laser beam focused onto a spot size of 10 μm by a 20× objective with numerical aperture = 0.4. The *V*_Si_ photoluminescence band centered around 917 nm was collected by the same objective, spectrally separated from the reflected laser beam using an 805-nm dichroic mirror and an 850-nm long-pass filter, and detected by a silicon photodiode. The output signal of the photodiode was locked in to the amplitude-modulation frequency of the RF signal applied to the IDT.

## Supplementary Materials

### This PDF file includes:

Sections S1 to S4 Figs. S1 to S3 References
