# Supplementary material for: Acoustically induced coherent spin trapping
Source: Sci Adv. 2021 Oct 29;7(44):eabj5030. doi: 10.1126/sciadv.abj5030 (PMC8555898; doi:10.1126/sciadv.abj5030)
Supplement: Supplementary file 1 — Sections S1 to S4 Figs. S1 to S3 References [file sciadv.abj5030_sm.pdf]

Science Advances
AAAS

Supplementary Materials for

Acoustically induced coherent spin trapping

Alberto Hernández-Mínguez*, Alexander V. Poshakinskiy, Michael Hollenbach, Paulo V. Santos, Georgy V. Astakhov

*Corresponding author. Email: alberto.h.minguez@pdi-berlin.de

Published 29 October 2021, *Sci. Adv.* **7**, eabj5030 (2021)
DOI: 10.1126/sciadv.abj5030

This PDF file includes:

Sections S1 to S4
Figs. S1 to S3
References

S1. CHARACTERIZATION OF THE ACOUSTIC RESONATOR

Figure S1a displays the RF power scattering (S) parameters of IDT1 and IDT2 in the acoustic resonator, measured at room temperature with a vector network analyser. The reflection coefficients (S_{11}, S_{22}) show a series of dips within the resonance band of the IDTs, accompanied by a series of peaks in the transmission coefficient (S_{21}). Such dips/peaks correspond to the excitation of the Rayleigh SAW modes in the resonator. Figure S1b displays the temperature dependence of the S_{11} reflection coefficient. The SAW resonances move towards higher frequencies as the temperature decreases. This blue shift was taken into account in the measurements as a function of temperature in the manuscript.

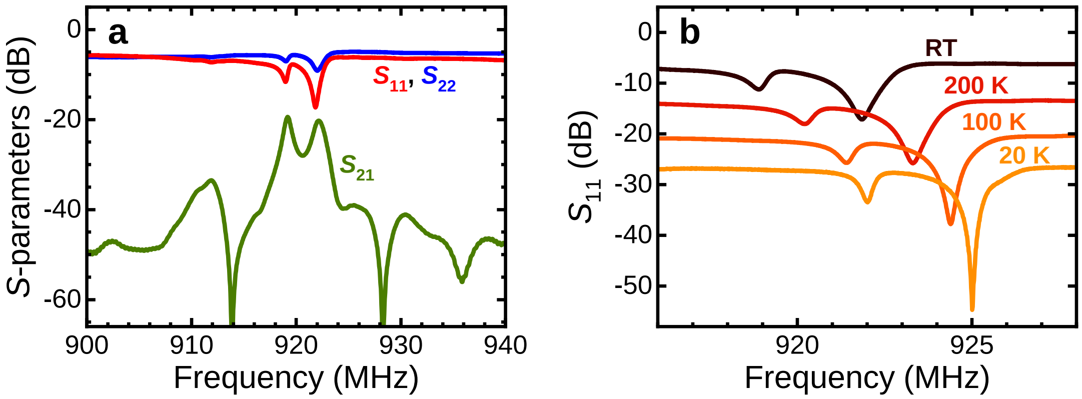

FIG. S1. **Characterization of the acoustic resonator. a,** Room-temperature power scattering parameters of the acoustic resonator as a function of the applied RF frequency. S_{11} and S_{22} are the power reflection coefficients of IDT1 and IDT2, respectively, while S_{21} is the power transmission coefficient. The S_{21} spectrum is time-gated to remove the electromagnetic cross-talk. **b,** Temperature dependence of the S_{11} coefficient. The curves are vertically shifted for clarity.

S2. THEORY OF COHERENT SPIN TRAPPING

To introduce the concept of coherent spin trapping (CST), we first consider a simplified model that is limited to one ground state (GS) and one excited state (ES). The full model used to describe the experimental optically detected magnetic resonance (ODMR) spectra features additionally an intermediate metastable state (MS) that is necessary

* alberto.h.minguez@pdi-berlin.de

to explain the optical pump and read-out of the spin polarization, see Sec. S3. We limit the consideration to just two spin sublevels in the GS and two spin sublevels in the ES. Then, the state of the system is described by the spin density matrices 2×2 of the GS and ES,

$$\rho_g = \frac{1}{2} N_g + \boldsymbol{S}_g \cdot \boldsymbol{\sigma}, \tag{S1}$$

$$\rho_e = \frac{1}{2} N_e + \boldsymbol{S}_e \cdot \boldsymbol{\sigma}, \tag{S2}$$

where N_g and N_e are the occupancies of the GS and ES, while $\boldsymbol{S}_g$ and $\boldsymbol{S}_e$ are the pseudospins of the GS and ES. Optical transitions between GS and ES are supposed to be incoherent and described by the rate equation. Within the GS and the ES, the evolution of the spin is assumed to be coherent; when the system goes from the GS to the ES and vice versa, spin coherence is assumed to be preserved. The dynamical equations read:

$$\frac{d\rho_g}{dt} = \frac{\mathrm{i}}{\hbar}[\rho_g, H_g] + \Gamma\rho_e - P\rho_g, \tag{S3}$$

$$\frac{d\rho_e}{dt} = \frac{\mathrm{i}}{\hbar}[\rho_e, H_e] - \Gamma\rho_e + P\rho_g, \tag{S4}$$

where

$$H_{g,e} = (\hbar/2)\boldsymbol{\Omega}^{(g,e)}(t) \cdot \boldsymbol{\sigma} \tag{S5}$$

are the Hamiltonians in the GS and ES, P is the optical pumping rate, and Γ is the relaxation rate of the ES. The spin and population dynamics are described by the decoupled equations:

$$\frac{dN_g}{dt} = \Gamma N_e - PN_g, \tag{S6}$$

$$\frac{dN_e}{dt} = -\Gamma N_e + PN_g, \tag{S7}$$

and

$$\frac{d\boldsymbol{S}_g}{dt} = \boldsymbol{\Omega}^{(g)}(t) \times \boldsymbol{S}_g + \Gamma\boldsymbol{S}_e - P\boldsymbol{S}_g - \gamma\boldsymbol{S}_g + \Sigma\hat{\boldsymbol{z}}, \tag{S8}$$

$$\frac{d\boldsymbol{S}_e}{dt} = \boldsymbol{\Omega}^{(e)}(t) \times \boldsymbol{S}_e - \Gamma\boldsymbol{S}_e + P\boldsymbol{S}_g - \gamma\boldsymbol{S}_e, \tag{S9}$$

where we introduced the spin relaxation rate γ and the phenomenological spin pumping rate Σ (the origin of the spin pumping is the relaxation via a MS, as described in Sec. S3).

To calculate the spin resonance spectra, we take

$$\boldsymbol{\Omega}^{(g,e)}(t) = \Omega_z^{(g,e)}\hat{\boldsymbol{z}} + \Omega_{\mathrm{rf}}^{(g,e)}(t), \tag{S10}$$

where

$$\Omega_z^{(g,e)} = \Delta E^{(g,e)}/\hbar \tag{S11}$$

are the Zeeman splittings of the considered spin state pairs in the GS and ES, and

$$\Omega_{\mathrm{rf}}^{(g,e)}(t) = \mathrm{Re}\,\Omega_R^{(g,e)}(\hat{\boldsymbol{x}} + \mathrm{i}\hat{\boldsymbol{y}})\mathrm{e}^{-\mathrm{i}\omega t} \tag{S12}$$

is the driving field. The coordinate frame (x, y, z) used here corresponds to the frame (x', y', z') in the Introduction of the main text. For simplicity, we assume that the driving field is circularly polarized, and switch to the reference frame rotating with its frequency ω. There, the spin dynamics is governed by the static effective field

$$\tilde{\boldsymbol{\Omega}}^{(g,e)} = (\Omega_z^{(g,e)} - \omega)\hat{\boldsymbol{z}} + \Omega_R^{(g,e)}\hat{\boldsymbol{x}}. \tag{S13}$$

The normalized spin-resonance (SR) signal is found as

$$\mathrm{R} = 1 - \gamma(S_{g,z}^{(0)} + S_{e,z}^{(0)})/\Sigma, \tag{S14}$$

where $S_{g,e}^{(0)}$ are the steady-state solutions of Eqs. (S8)-(S9) with $\mathbf{\Omega}^{(g,e)}(t)$ replaced by $\tilde{\mathbf{\Omega}}^{(g,e)}$, Eq. (S13).

First, we consider the limit $|\Omega_z^{(e)} - \Omega_z^{(g)}| \to \infty$, that is, when the spin resonances corresponding to the GS and ES are well separated spectrally. The SR signal in the vicinity of each resonance is then given by

$$\mathrm{R}_g(\omega) = \frac{\frac{P\Gamma}{\Gamma+P}\frac{\Omega_R^{(g)\,2}}{\gamma}}{(\Omega_z^{(g)} - \omega)^2 + P^2 + \frac{P\Gamma}{\Gamma+P}\frac{\Omega_R^{(g)\,2}}{\gamma}}\,, \tag{S15}$$

$$\mathrm{R}_e(\omega) = \frac{\frac{P\Gamma}{\Gamma+P}\frac{\Omega_R^{(e)\,2}}{\gamma}}{(\Omega_z^{(e)} - \omega)^2 + \Gamma^2 + \frac{P\Gamma}{\Gamma+P}\frac{\Omega_R^{(e)\,2}}{\gamma}}\,, \tag{S16}$$

where we have assumed $\Gamma \sim P \gg \gamma$. The area below the isolated GS and ES state resonances is related as

$$\frac{\int \mathrm{R}_g(\omega)\,d\omega}{\int \mathrm{R}_e(\omega)\,d\omega} = \frac{\Omega_R^{(g)^2}/\gamma}{\Omega_R^{(e)^2}/\gamma}\,\frac{\Gamma}{P} \tag{S17}$$

where the first factor in the right-hand side is the ratio of spin transition intensities in the GS and ES, the second factor is the ratio of times the center spends in GS and ES, and we assumed the limit of small $\Omega_R^{(g)}$ and $\Omega_R^{(e)}$. In the experiment, the ratio of the peak areas was found to be ≈ 50 in the case of weak optical pumping $P \lesssim \Gamma$. Therefore, we conclude that the intensity of the spin transitions in the ES more than 50 times stronger than in the GS.

When the resonances corresponding to the GS and ES overlap, then the SR signal reads

$$\mathrm{R} = \frac{\Gamma P}{\gamma(\Gamma+P)}\,\frac{[(\Omega_z^{(g)}-\omega)\Omega_R^{(e)} - (\Omega_z^{(e)}-\omega)\Omega_R^{(g)}]^2}{[\Gamma(\Omega_z^{(g)}-\omega) + P(\Omega_z^{(e)}-\omega)]^2 + (\Omega_z^{(g)}-\omega)^2(\Omega_z^{(e)}-\omega)^2 + [(\Omega_z^{(g)}-\omega)\Omega_R^{(e)} - (\Omega_z^{(e)}-\omega)\Omega_R^{(g)}]^2}. \tag{S18}$$

Note that the SR signal vanishes if

$$\frac{\Omega_z^{(g)} - \omega}{\Omega_z^{(e)} - \omega} = \frac{\Omega_R^{(g)}}{\Omega_R^{(e)}}, \tag{S19}$$

i.e., when the total precession frequencies in the GS and ES are collinear. This is realized at

$$\omega_{\mathrm{CST}} = \frac{\Omega_z^{(g)}\Omega_R^{(e)} - \Omega_z^{(e)}\Omega_R^{(g)}}{\Omega_R^{(e)} - \Omega_R^{(g)}}. \tag{S20}$$

Under this condition, the spin precesses around the same direction in the GS and ES. As a consequence, the spin projection along this direction does not dephase under the random switching between the GS and ES, but conserves its coherence on the long time scale $1/\gamma$. This situation is what we define as CST. Away from the CST condition, the precession frequency and direction change continuously as the centre switches randomly between the GS and ES. This causes the spin to dephase with the rate $\sim [(\Omega_z^{(e)} - \Omega_z^{(g)})^2 + (\Omega_R^{(e)} - \Omega_R^{(g)})^2]/(\Gamma + P)$, á la Dyakonov–Perel spin relaxation mechanism.

The angle between the trapped spin component and z axis is given by

$$\theta_{\mathrm{CST}} = \frac{\Omega_R^{(e)} - \Omega_R^{(g)}}{\Omega_z^{(e)} - \Omega_z^{(g)}}\,. \tag{S21}$$

The SR signal at CST is given by

$$\mathrm{R}(\omega_{\mathrm{CST}}) = 1 - \cos^2\theta_{\mathrm{CST}} = \frac{(\Omega_R^{(e)} - \Omega_R^{(g)})^2}{(\Omega_z^{(e)} - \Omega_z^{(g)})^2 + (\Omega_R^{(e)} - \Omega_R^{(g)})^2}\,. \tag{S22}$$

At small Rabi frequencies, this value is small, so CST is revealed as a sharp spectral dip.

S3. OPTICALLY DETECTED SPIN TRAPPING

To describe the manifestation of the CST in the spectra of the ODMR, we consider a model that accounts for the GS, ES and intermediate MS, see Fig. S2. Only two spin sublevels of each level are considered (pseudospin 1/2). To explain optical orientation (appearance of polarization under non-resonant optical pumping) and read-out (variation of the photoluminescence (PL) intensity depending of the spin polarization), the ES to MS relaxation is assumed to be spin dependent (parameter η). The model is described by the equation set for the spin-density matrices ρ_g, ρ_e, and ρ_m for GS, ES, and MS:

$$\frac{d\rho_e}{dt} = \frac{\mathrm{i}}{\hbar}[\rho_e, H_e] - \Gamma\rho_e - \Gamma_{m1}\{(1+\eta\sigma_z), \rho_e\} + P\rho_g \,, \tag{S23}$$

$$\frac{d\rho_g}{dt} = \frac{\mathrm{i}}{\hbar}[\rho_g, H_g] + \Gamma\rho_e - P\rho_g + \Gamma_{m2}\rho_m \,, \tag{S24}$$

$$\frac{d\rho_m}{dt} = \frac{\mathrm{i}}{\hbar}[\rho_m, H_m] + \Gamma_{m1}\{(1+\eta\sigma_z), \rho_e\} - \Gamma_{m2}\rho_m \,. \tag{S25}$$

This yields the coupled equations for the populations

$$\frac{dN_e}{dt} = -\Gamma N_e - \Gamma_{m1}(N_e + 2\eta S_e^z) + PN_g \,, \tag{S26}$$

$$\frac{dN_g}{dt} = \Gamma N_e - PN_g + \Gamma_{m2}N_m \,, \tag{S27}$$

$$\frac{dN_m}{dt} = \Gamma_{m1}(N_e + 2\eta S_e^z) - \Gamma_{m2}N_m \,, \tag{S28}$$

and spins

$$\frac{d\boldsymbol{S}_e}{dt} = \boldsymbol{\Omega}^{(e)} \times \boldsymbol{S}_e - \gamma_e\boldsymbol{S}_e - \Gamma\boldsymbol{S}_e - \Gamma_{m1}(\boldsymbol{S}_e + \tfrac{1}{2}\eta N_e\boldsymbol{e}^z) + P\boldsymbol{S}_g \,, \tag{S29}$$

$$\frac{d\boldsymbol{S}_g}{dt} = \boldsymbol{\Omega}^{(g)} \times \boldsymbol{S}_g - \gamma_g\boldsymbol{S}_g + \Gamma\boldsymbol{S}_e - P\boldsymbol{S}_g + \Gamma_{m2}\boldsymbol{S}_m \,, \tag{S30}$$

$$\frac{d\boldsymbol{S}_m}{dt} = \boldsymbol{\Omega}^{(m)} \times \boldsymbol{S}_m - \gamma_m\boldsymbol{S}_m + \Gamma_{m1}(\boldsymbol{S}_e + \tfrac{1}{2}\eta N_e\boldsymbol{e}^z) - \Gamma_{m2}\boldsymbol{S}_m \,. \tag{S31}$$

The PL intensity is given by

$$I_{\mathrm{PL}} = \Gamma N_e \,. \tag{S32}$$

We assume $\eta \ll 1$ and evaluate PL up to η^2. Neglecting η, the steady-state ES population reads

$$N_e^{(0)} = \frac{\Gamma_{m2}P}{\Gamma_{m1}(\Gamma_{m2}+P) + \Gamma_{m2}(\Gamma+P)} \,. \tag{S33}$$

Spin-dependent relaxation from ES to MS leads to the generation of the spin in the ES and MS which is described by $\boldsymbol{G}_e = -\frac{1}{2}\eta\Gamma_{m1}N_e^{(0)}\hat{\boldsymbol{z}}$, $\boldsymbol{G}_g = 0$, $\boldsymbol{G}_m = \frac{1}{2}\eta\Gamma_{m1}N_e^{(0)}\hat{\boldsymbol{z}}$, and the following steady-state spin polarizations (up to terms

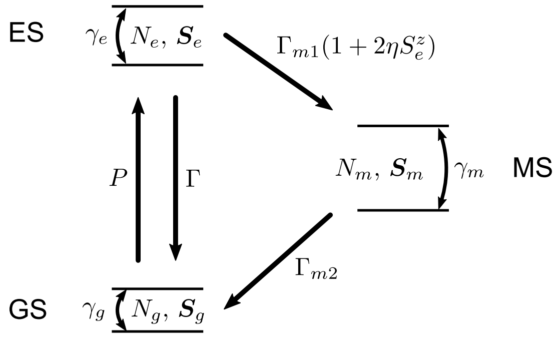

FIG. S2. The three-level model of the spin centre.

linear in η):

$$\begin{pmatrix} S_e^z \\ S_g^z \\ S_m^z \end{pmatrix} = \begin{pmatrix} -(\gamma_g\Gamma_{m2} + \gamma_m P + \gamma_g\gamma_m) \\ \gamma_e\Gamma_{m2} - \gamma_m\Gamma \\ \gamma_g\Gamma + \gamma_e P + \gamma_g\gamma_e \end{pmatrix}$$
$$\times \frac{\frac{1}{2}\eta N_e^{(0)}\Gamma_{m1}}{\gamma_g\gamma_m(\Gamma+\gamma_e+\Gamma_{m1}) + \gamma_g\Gamma_{m2}(\Gamma+\gamma_e+\Gamma_{m1}) + \gamma_m P(\gamma_e+\Gamma_{m1}) + \gamma_e\Gamma_{m2}P} . \tag{S34}$$

Note that the GS and ES have opposite spin orientation if

$$\frac{\Gamma_{m2}}{\gamma_m} > \frac{\Gamma}{\gamma_e} . \tag{S35}$$

This condition means that the spin coming to the GS from the MS ($\propto \Gamma_{m2}/\gamma_m$) dominates over the spin coming to the GS from the ES ($\propto \Gamma/\gamma_e$). If condition (S35) is fulfilled, the ODMR signal is negative for the ES and positive for the GS (as observed in the experiment). If the condition (S35) is not fulfilled, then the ODMR signal is negative for both ES and GS. For simplicity, we assume in the following that $\gamma_m = 0$, thus fulfilling the condition (S35). We also assume that the MS is far off-resonance, $\Omega_z^{(m)} \to \infty$, which corresponds to $S_m^x, S_m^y = 0$. Then, up to terms quadratic in $\Omega_R^{(g)}$ and $\Omega_R^{(e)}$, the ODMR signal reads

$$\Delta\mathrm{PL}/\mathrm{PL} \propto \tag{S36}$$
$$\frac{\eta^2}{(\gamma_g(\Gamma+\gamma_e)+\gamma_e P)^2 \left(\Delta\omega_e^2\left(\Delta\omega_g^2 + (\gamma_g+W_g+P)^2\right) + 2\Delta\omega_e\Delta\omega_g\Gamma P + \Delta\omega_g^2(\Gamma+\gamma_e)^2 + ((\Gamma+\gamma_e)(\gamma_g+W_g)+\gamma_e P)^2\right)}$$
$$\times \Big[\gamma_e P\Omega_R^{(g)2}\left(\Delta\omega_e^2(\gamma_g+W_g+P) + (\Gamma+\gamma_e)((\Gamma+\gamma_e)(\gamma_g+W_g)+\gamma_e P)\right)$$
$$+ P\Omega_R^{(g)}\Omega_R^{(e)}(\gamma_e(\gamma_g+P) - \Gamma\gamma_g)(-\Delta\omega_e\Delta\omega_g + (\Gamma+\gamma_e)(\gamma_g+W_g)+\gamma_e P)$$
$$- \gamma_g\Omega_R^{(e)2}(\gamma_g+P)\left(\Delta\omega_g^2(\Gamma+\gamma_e) + (\gamma_g+W_g+P)((\Gamma+\gamma_e)(\gamma_g+W_g)+\gamma_e P)\right)\Big] ,$$

where $\Delta\omega_{g,e} = \Omega_z^{(g,e)} - \omega$ and we supposed $\Gamma_{m1}, \Gamma_{m2} \to 0$ for simplicity. We also introduced the inhomogeneous GS resonance broadening W_g.

A. Resonance width

When the GS and ES resonances occur as close frequencies, the wide ES resonance remains almost unaffected, while the shape of the narrower GS resonance can change significantly. In vicinity of the GS resonance, $\omega \approx \Omega_z^{(g)}$, Eq. (S36) can be rewritten in the form [cf. Eq. (4) of the main text]

$$\Delta\mathrm{PL}/\mathrm{PL} = \frac{A_g\Gamma_g^2 + B_g\Gamma_g\widetilde{\Delta\omega}_g}{\widetilde{\Delta\omega}_g^2 + \Gamma_g^2} \tag{S37}$$

where $\widetilde{\Delta\omega}_g = \Delta\omega_g - \delta_g$ is the detuning from the GS resonance, which appears to be slightly shifted by

$$\delta_g = -\frac{(\Omega_z^{(e)} - \Omega_z^{(g)})\Gamma P}{(\Omega_z^{(e)} - \Omega_z^{(g)})^2 + (\Gamma+\gamma_e+\Gamma_{m1})^2} , \tag{S38}$$

A_g and B_g are the amplitudes of the symmetric and anti-symmetric parts of the resonance, and Γ_g is the resonance width. The latter reads

$$\Gamma_g = \gamma_g + P\left(1 - \frac{\Gamma(\Gamma+\Gamma_{m1}+\gamma_e)}{(\Omega_z^{(e)} - \Omega_z^{(g)})^2 + (\Gamma+\Gamma_{m1}+\gamma_e)^2}\right) + W_g . \tag{S39}$$

When the GS and ES resonances are well separated, $|\Omega_z^{(e)} - \Omega_z^{(g)}| \to \infty$, the width of the GS resonance is

$$\Gamma_g^{(0)} = \gamma_g + P + W_g , \tag{S40}$$

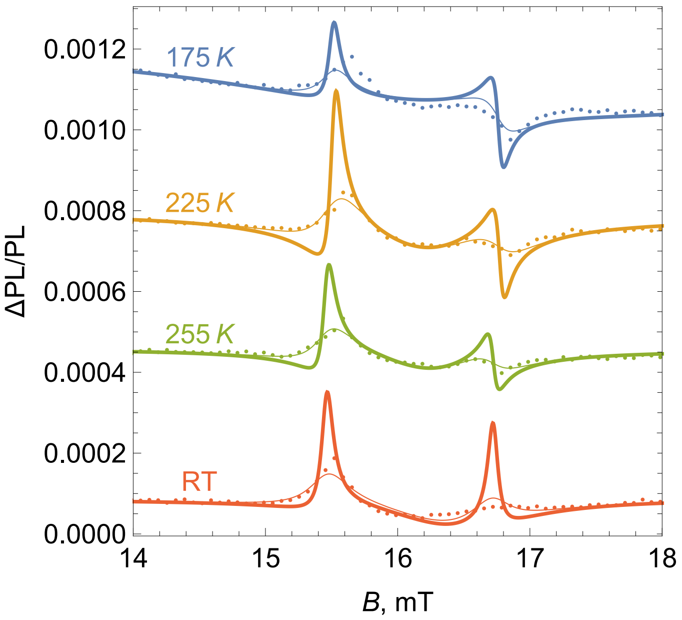

FIG. S3. **Experimental ODMR spectra (points) and the result of their fitting with Eq.** (S36) **(thin lines).** Thick lines show the calculation made for a smaller value of the inhomogeneous broadening $W_g = 2.5\,\text{MHz}$, while all other parameters remain the same as in the fit.

i.e., is determined by the sum of spin relaxation rate in the GS γ_g, the rate of the optical transitions from the GS to the ES P, and the inhomogeneous broadening W_g. As the two resonances come close to each other, the width decreases according to Eq. (S39). Namely, the contribution $\propto P$ gets suppressed, which can be interpreted as a result of CST: the spin dynamics becomes immune to optical transitions. When the GS and ES resonances coincide, $\Omega_z^{(e)} = \Omega_z^{(g)}$, the smallest resonance width is achieved,

$$\Gamma_g^{(\text{CST})} = \gamma_g + \frac{\Gamma_{m1} + \gamma_e}{\Gamma + \Gamma_{m1} + \gamma_e} P + W_g\,. \tag{S41}$$

Note that the pump-induced broadening P is strongly suppressed by a factor $(\Gamma_{m1} + \gamma_e)/(\Gamma + \Gamma_{m1} + \gamma_e) \ll 1$ for the relevant case $\Gamma_{m1}, \gamma_e \ll \Gamma$ [38] (see parameters in the Table in Sec. S4). Unfortunately, in our sample, the main contribution to the resonance width is given by the strong inhomogeneous broadening W_g which does not allow us to resolve the homogeneous linewidth variation. Observation of the CST induced line narrowing could be done using single spin defects, which is out of scope of the present manuscript.

S4. FIT OF EXPERIMENTAL ODMR SPECTRA

To model the ODMR spectra, we used a sum of two signals, corresponding to the $-3/2 \leftrightarrow +1/2$ and $+3/2 \leftrightarrow -1/2$ transitions. At each temperature, we used $\Omega_z^{(g,e)}$ equal to the spin splitting of the corresponding states, and all other parameters were the same for both signals:

T, K	$\Omega_R^{(e)}/\Omega_R^{(g)}$, 10^2	P, MHz	Γ, MHz	W_g, MHz	γ_g, MHz	γ_e, MHz
175	-4.6	0.38	86	7.7	0.4×10^{-4}	0.4
225	-4.9	0.65	260	10.7	1.2×10^{-4}	1.2
255	-3.5	0.76	240	9.3	2.1×10^{-4}	2.1
300	-1.2	1	250	10	4.6×10^{-4}	4.6

Since the intensity of the spin transitions in the ES and GS is in fact determined by the combination of parameters $\Omega_R^{(g,e)2}/\gamma_{g,e}$, there is an ambiguity in their separate determination from the fit. To be specific, we used here γ_g from the temperature dependence of the spin relaxation rate measured in Ref. 39; the ratio of the spin-acoustic interaction

constants in ES and GS $\Omega_R^{(e)}/\Omega_R^{(g)}$ was supposed to be $\sim 10^2$ as suggested by preliminary experimental data on the value of the spin-strain interaction constant; in accordance, the spin relaxation in the ES was supposed to be much faster than in the GS $\gamma_e = 10^4\,\gamma_g$. The other parameters were fitted to obtain the best match with the experimental data. The result of the fits are shown as thin curves in Fig. S3. For comparison, we also show as thick curves the same ODMR spectra, but with a smaller inhomogeneous broadening $W_g = 2.5\,\mathrm{MHz}$. In this case, the amplitude of the resonances increases and their asymmetry becomes more pronounced.

REFERENCES AND NOTES

1. O. Arcizet, V. Jacques, A. Siria, P. Poncharal, P. Vincent, S. Seidelin, A single nitrogen-vacancy defect coupled to a nanomechanical oscillator. *Nat. Phys.* **7**, 879–883 (2011).

2. S. Kolkowitz, A. C. Bleszynski Jayich, Q. P. Unterreithmeier, S. D. Bennett, P. Rabl, J. G. E. Harris, M. D. Lukin, Coherent sensing of a mechanical resonator with a single-spin qubit. *Science* **335**, 1603–1606 (2012).

3. A. Barfuss, J. Teissier, E. Neu, A. Nunnenkamp, P. Maletinsky, Strong mechanical driving of a single electron spin. *Nat. Phys.* **11**, 820–824 (2015).

4. M. J. A. Schuetz, E. M. Kessler, G. Giedke, L. M. K. Vandersypen, M. D. Lukin, J. I. Cirac, Universal quantum transducers based on surface acoustic waves. *Phys. Rev. X* **5**, 031031 (2015).

5. E. R. MacQuarrie, M. Otten, S. K. Gray, G. D. Fuchs, Cooling a mechanical resonator with nitrogen-vacancy centres using a room temperature excited state spin–strain interaction. *Nat. Commun.* **8**, 14358 (2017).

6. D. Lee, K. W. Lee, J. V. Cady, P. Ovartchaiyapong, A. C. B. Jayich, Topical review: Spins and mechanics in diamond. *J. Optics* **19**, 033001 (2017).

7. M.-A. Lemonde, S. Meesala, A. Sipahigil, M. J. A. Schuetz, M. D. Lukin, M. Loncar, P. Rabl. Phonon networks with silicon-vacancy centers in diamond waveguides. *Phys. Rev. Lett.* **120**, 213603 (2018).

8. B. Machielse, S. Bogdanovic, S. Meesala, S. Gauthier, M. J. Burek, G. Joe, M. Chalupnik, Y. I. Sohn, J. Holzgrafe, R. E. Evans, C. Chia, H. Atikian, M. K. Bhaskar, D. D. Sukachev, L. Shao, S. Maity, M. D. Lukin, M. Lončar. Quantum interference of electromechanically stabilized emitters in nanophotonic devices. *Phys. Rev. X* **9**, 031022 (2019).

9. A. V. Poshakinskiy, G. V. Astakhov. Optically detected spin-mechanical resonance in silicon carbide membranes. *Phys. Rev. B* **100**, 094104 (2019).

10. H. Wang, I. Lekavicius. Coupling spins to nanomechanical resonators: Toward quantum spin-mechanics. *Appl. Phys. Lett.* **117**, 230501 (2020).

11. P. Delsing, A. N. Cleland, M. J. A. Schuetz, J. Knörzer, G. Giedke, J. I. Cirac, K. Srinivasan, M. Wu, K. C. Balram, C. Bäuerle, T. Meunier, C. J. B. Ford, P. V. Santos, E. Cerda-Méndez, H. Wang, H. J. Krenner, E. D. S. Nysten, M. Weiß, G. R. Nash, L. Thevenard, C. Gourdon, P. Rovillain, M. Marangolo, J.-Y. Duquesne, G. Fischerauer, W. Ruile, A. Reiner, B. Paschke, D. Denysenko, D. Volkmer, A. Wixforth, H. Bruus, M. Wiklund, J. Reboud, J. M. Cooper, Y. Q. Fu, M. S. Brugger, F. Rehfeldt, C. Westerhausen, The 2019 surface acoustic waves roadmap. *J. Phys. D Appl. Phys.* **52**, 353001 (2019).

12. S. J. Whiteley, G. Wolfowicz, C. P. Anderson, A. Bourassa, H. Ma, M. Ye, G. Koolstra, K. J. Satzinger, M. V. Holt, F. J. Heremans, A. N. Cleland, D. I. Schuster, G. Galli, D. D. Awschalom, Spin–phonon interactions in silicon carbide addressed by Gaussian acoustics. *Nat. Phys.* **15**, 490–495 (2019).

13. S. Maity, L. Shao, S. Bogdanović, S. Meesala, Y.-I. Sohn, N. Sinclair, B. Pingault, M. Chalupnik, C. Chia, L. Zheng, K. Lai, M. Lončar, Coherent acoustic control of a single silicon vacancy spin in diamond. *Nat. Commun.* **11**, 193 (2020).

14. A. Hernández-Mínguez, A. V. Poshakinskiy, M. Hollenbach, P. V. Santos, G. V. Astakhov. Anisotropic spin-acoustic resonance in silicon carbide at room temperature. *Phys. Rev. Lett.* **125**, 107702 (2020).

15. P. Udvarhelyi, G. Thiering, N. Morioka, C. Babin, F. Kaiser, D. Lukin, T. Ohshima, J. Ul-Hassan, N. T. Son, J. Vučković, J. Wrachtrup, A. Gali. Vibronic states and their effect on the temperature and strain dependence of silicon-vacancy qubits in 4*H*-SiC. *Phys. Rev. Appl.* **13**, 054017 (2020).

16. A. N. Anisimov, D. Simin, V. A. Soltamov, S. P. Lebedev, P. G. Baranov, G. V. Astakhov, V. Dyakonov, Optical thermometry based on level anticrossing in silicon carbide. *Sci. Rep.* **6**, 33301 (2016).

17. D. M. Lukin, A. D. White, R. Trivedi, M. A. Guidry, N. Morioka, C. Babin, Ö. O. Soykal, J. Ul-Hassan, N. T. Son, T. Ohshima, P. K. Vasireddy, M. H. Nasr, S. Sun, J.-P. W. MacLean, C. Dory, E. A. Nanni, J. Wrachtrup, F. Kaiser, J. Vučković, Spectrally reconfigurable quantum emitters enabled by optimized fast modulation. *npj Quantum Inf.* **6**, 80 (2020).

18. D. A. Golter, T. Oo, M. Amezcua, K. A. Stewart, H. Wang, Optomechanical quantum control of a nitrogen-vacancy center in diamond. *Phys. Rev. Lett.* **116**, 143602 (2016).

19. D. A. Golter, T. Oo, M. Amezcua, I. Lekavicius, K. A. Stewart, H. Wang, Coupling a surface acoustic wave to an electron spin in diamond via a dark state. *Phys. Rev. X* **6**, 041060 (2016).

20. M. Fleischhauer, A. Imamoglu, J. P. Marangos, Electromagnetically induced transparency: Optics in coherent media. *Rev. Mod. Phys.* **77**, 633–673 (2005).

21. A. Abragam, *The Principles of Nuclear Magnetism* (Clarendon Press, Oxford, 1961).

22. M. I. Dyakonov, V. I. Perel, Spin relaxation of conduction electrons in noncentrosymmetric semiconductors. *Fiz. Tverd. Tela* **13**, 3581 (1971).

23. A. V. Shumilin, V. V. Kabanov. Kinetic equations for hopping transport and spin relaxation in a random magnetic field. *Phys. Rev. B* **92**, 014206 (2015).

24. A. V. Shumilin, E. Y. Sherman, M. M. Glazov. Spin dynamics of hopping electrons in quantum wires: Algebraic decay and noise. *Phys. Rev. B* **94**, 125305 (2016).

25. R. Kubo. Note on the stochastic theory of resonance absorption. *J. Physical Soc. Japan* **9**, 935 (1954), 944.

26. R. Nagy, M. Niethammer, M. Widmann, Y.-C. Chen, P. Udvarhelyi, C. Bonato, J. Ul Hassan, R. Karhu, I. G. Ivanov, N. T. Son, J. R. Maze, T. Ohshima, Ö. O. Soykal, Á. Gali, S.-Y. Lee, F. Kaiser, J. Wrachtrup, High-fidelity spin and optical control of single silicon-vacancy centres in silicon carbide. *Nat. Commun.* **10**, 1954 (2019).

27. I. D. Breev, A. V. Poshakinskiy, V. V. Yakovleva, S. S. Nagalyuk, E. N. Mokhov, R. Hübner, G. V. Astakhov, P. G. Baranov, A. N. Anisimov, Stress-controlled zero-field spin splitting in silicon carbide. *Appl. Phys. Lett.* **118**, 084003 (2021).

28. H. Kraus, V. A. Soltamov, D. Riedel, S. Väth, F. Fuchs, A. Sperlich, P. G. Baranov, V. Dyakonov, G. V. Astakhov, Room-temperature quantum microwave emitters based on spin defects in silicon carbide. *Nat. Phys.* **10**, 157–162 (2014).

29. H. Kraus, D. Simin, C. Kasper, Y. Suda, S. Kawabata, W. Kada, T. Honda, Y. Hijikata, T. Ohshima, V. Dyakonov, G. V. Astakhov, Three-dimensional proton beam writing of optically active coherent vacancy spins in silicon carbide. *Nano Lett.* **17**, 2865–2870 (2017).

30. S. A. Tarasenko, A. V. Poshakinskiy, D. Simin, V. A. Soltamov, E. N. Mokhov, P. G. Baranov, V. Dyakonov, G. V.Astakhov, Spin and optical properties of silicon vacancies in silicon carbide—A review. *Physica Status Solidi B* **255**, 1700258 (2018).

31. D. Simin, V. A. Soltamov, A. V. Poshakinskiy, A. N. Anisimov, R. A. Babunts, D. O. Tolmachev, E. N. Mokhov, M. Trupke, S. A. Tarasenko, A. Sperlich, P. G. Baranov, V. Dyakonov, G. V. Astakhov, All-optical dc nanotesla magnetometry using silicon vacancy fine structure in isotopically purified silicon carbide. *Phys. Rev. X* **6**, 031014 (2016).

32. W. Dong, M. W. Doherty, S. E. Economou. Spin polarization through intersystem crossing in the silicon vacancy of silicon carbide. *Phys. Rev. B* **99**, 184102 (2019).

33. T. M. Hoang, H. Ishiwata, Y. Masuyama, Y. Yamazaki, K. Kojima, S.-Y. Lee, T. Ohshima, T. Iwasaki, D. Hisamoto, M. Hatano, Thermometric quantum sensor using excited state of silicon vacancy centers in 4H-SiC devices. *Appl. Phys. Lett.* **118**, 044001 (2021).

34. Ö. O. Soykal, P. Dev, S. E. Economou, Silicon vacancy center in 4H-SiC: Electronic structure and spin-photon interfaces. *Phys. Rev. B* **93**, 081207 (2016).

35. M. Limonov, M. Rybin, A. Poddubny, Y. Kivshar. Fano resonances in photonics. *Nat. Photonics* **11**, 543–554 (2017).

36. V. A. Soltamov, C. Kasper, A. V. Poshakinskiy, A. N. Anisimov, E. N. Mokhov, A. Sperlich, S. A. Tarasenko, P. G.Baranov, G. V. Astakhov, V. Dyakonov, Excitation and coherent control of spin qudit modes in silicon carbide at room temperature. *Nat. Commun.* **10**, 1678 (2019).

37. D. Riedel, F. Fuchs, H. Kraus, S. Väth, A. Sperlich, V. Dyakonov, A. A. Soltamova, P. G. Baranov, V. A. Ilyin, G. V. Astakhov, Resonant addressing and manipulation of silicon vacancy qubits in silicon carbide. *Phys. Rev. Lett.* **109**, 226402 (2012).

38. F. Fuchs, B. Stender, M. Trupke, D. Simin, J. Pflaum, V. Dyakonov, G. V. Astakhov, Engineering near-infrared single-photon emitters with optically active spins in ultrapure silicon carbide. *Nat. Commun.* **6**, 7578 (2015).

39. D. Simin, H. Kraus, A. Sperlich, T. Ohshima, G. V. Astakhov, V. Dyakonov, Locking of electron spin coherence above 20 ms in natural silicon carbide. *Phys. Rev. B* **95**, 161201(R) (2017).
